# Supplementary material for: Lineage marker synchrony in hematopoietic genealogies refutes the PU.1/GATA1 toggle switch paradigm
Source: Nat Commun. 2018 Jul 12;9:2697. doi: 10.1038/s41467-018-05037-3 (PMC6043612; doi:10.1038/s41467-018-05037-3)
Supplement: Supplementary file 1 — Supplementary Information [file 41467_2018_5037_MOESM1_ESM.pdf]

Lineage marker synchrony in hematopoietic genealogies refutes the PU.1/GATA1 toggle switch paradigm

Strasser et al.

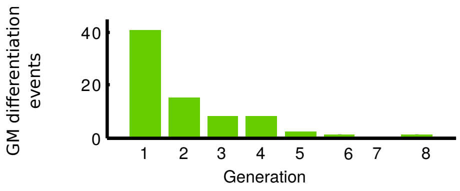

Supplementary Figure 1: Number of cells per predicted to differentiate to the GM-lineage decreases with generations.

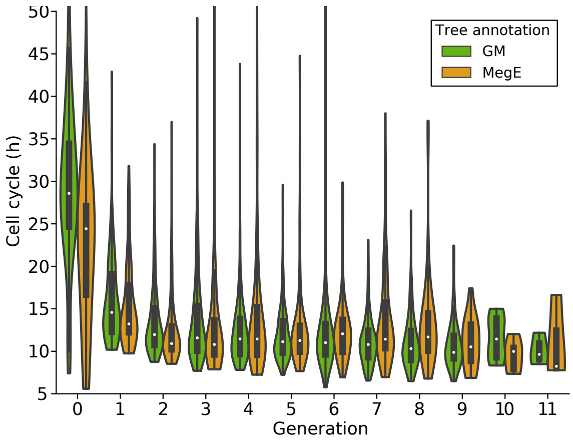

Supplementary Figure 2: Cell cycle decreases over generations. Data is stratified into trees with cells annotated as GM- or MegE-lineage exclusively. Note that cell cycle times in the first generation are censored as their "birth" happens before the movie start and is hence unobserved.

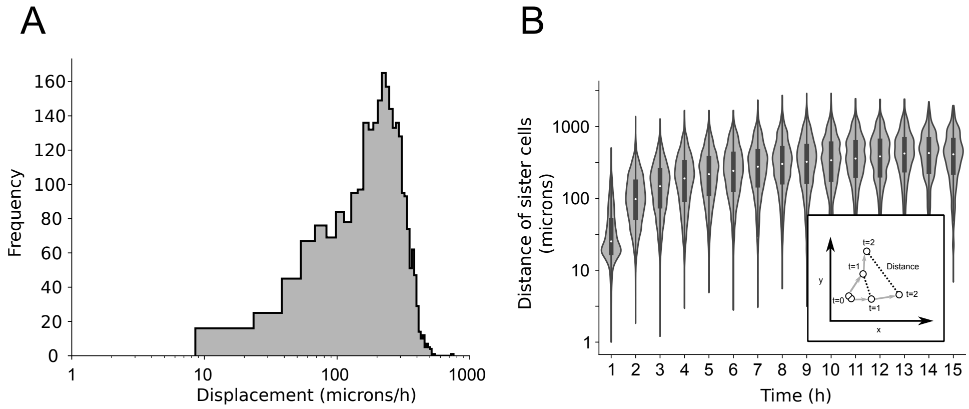

Supplementary Figure 3: Displacement of HSPCs. A) Distribution of HSPC displacement observed in the data from Hoppe et al. [1] (average speed 224 microns/h, $n = 2579$ cells, pooled from three experiments). As the average cell diameter is 14 microns, the majority of cells moves several cell diameters per hour. B) Distance between sister cells increases rapidly over time: A dividing cell gives rise to two sister cells in close proximity (see inset), which will rapidly move apart. At $t = 4h$ the median distance between all observed sisters is 190 microns).

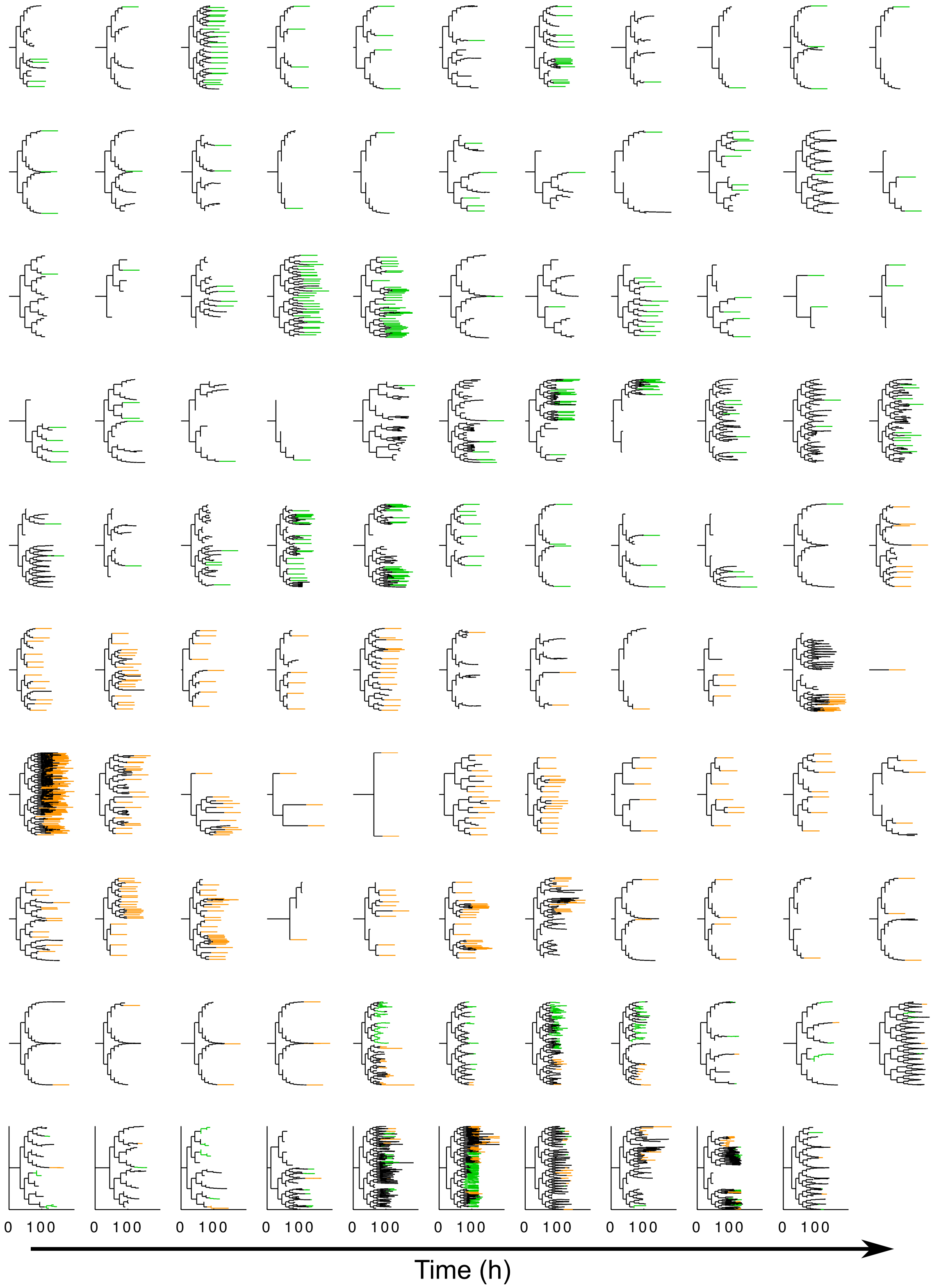

Supplementary Figure 4: Tracked GM, MegE, and GMMegE genealogies. CD16/32 (Gata1) onsets are indicated in green (orange).

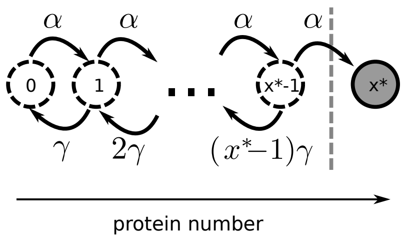

Supplementary Figure 5: Finite state projection for a simple gene expression model of the marker protein dynamics. Each node represents a state with protein number x. States are coupled via the corresponding synthesis and degradation reactions (corresponding reaction propensities are annotated at the arrows). The finite state projection is applied by truncating the protein state space at state $x^* - 1$ and introducing a new state x^*, that is absorbing (i.e. has no outgoing transitions). Therefore, all probability mass that moves beyond the border of the statespace accumulates in the state x^*.

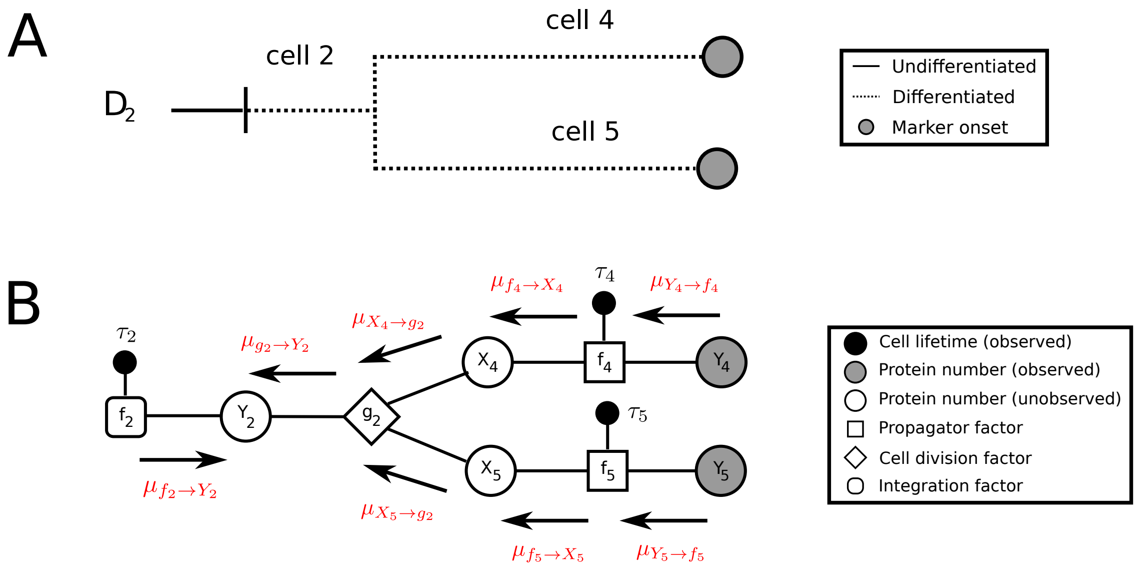

Supplementary Figure 6: Message passing on the graphical model is applied to obtain the likelihood of the tree shown in A. B) Messages are sent from the observed leaves (cells 4 and 5) towards the root (cell 2).

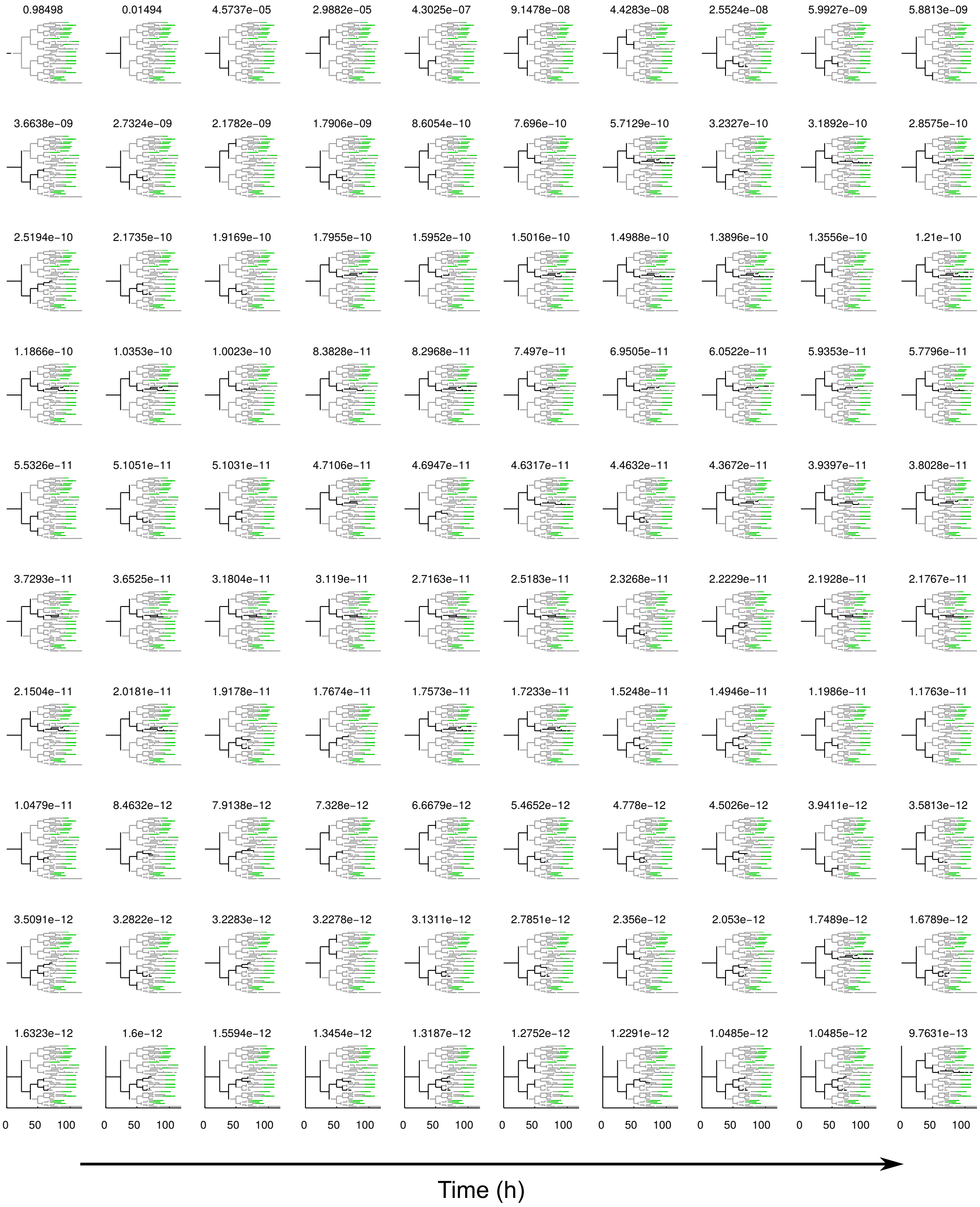

Supplementary Figure 7: Enumeration of the 100 most likely hidden trees corresponding to the first lineage tree shown in Fig. 3A (main text). The relative likelihoods $\frac{\mathcal{L}(H|\lambda(t),\alpha,\gamma,x^*)}{\sum_{H'}\mathcal{L}(H'|\lambda(t),\alpha,\gamma,x^*)}$ are indicated above each hidden tree and show strong evidence of lineage choice in the first generation.

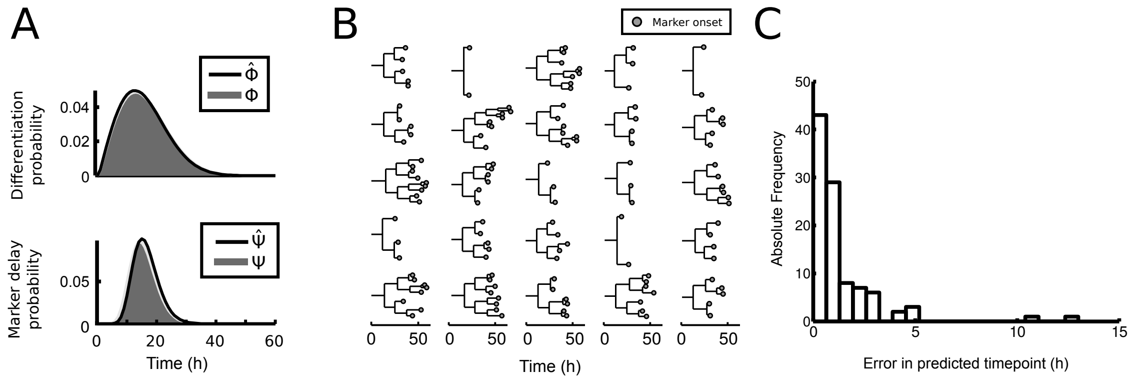

Supplementary Figure 8: Inference of lineage choice from trees can accurately reconstruct the underlying differentiation and marker delay dynamics. A,B) For a given set of parameters θ, η, the differentiation probability distribution Φ (gray, upper panel) and the marker delay probability distribution Ψ (gray, lower panel) are shown. We simulate 50 trees (25 shown in B) from these parameters and apply the tree inference algorithm to obtain estimates $\hat{\theta}, \hat{\eta}$ and distributions $\hat{\Phi}, \hat{\Psi}$ (solid lines), which match the true distributions. C) Histogram of the difference in time between predicted and true differentiation timepoints for an independent test set of 100 trees simulated from the true distributions (gray) in A.

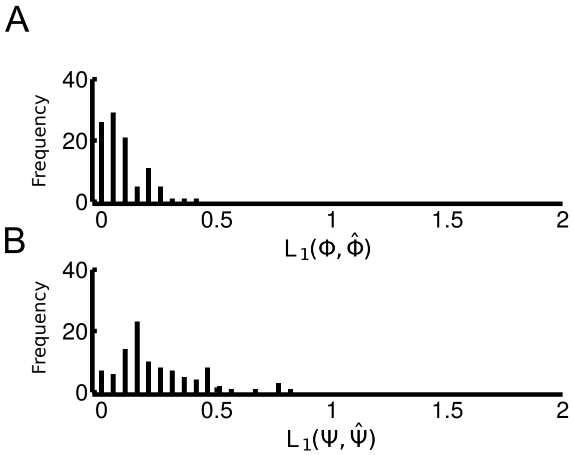

Supplementary Figure 9: L_1 distances between estimated and true differentiation (A) and delay (B) distributions.

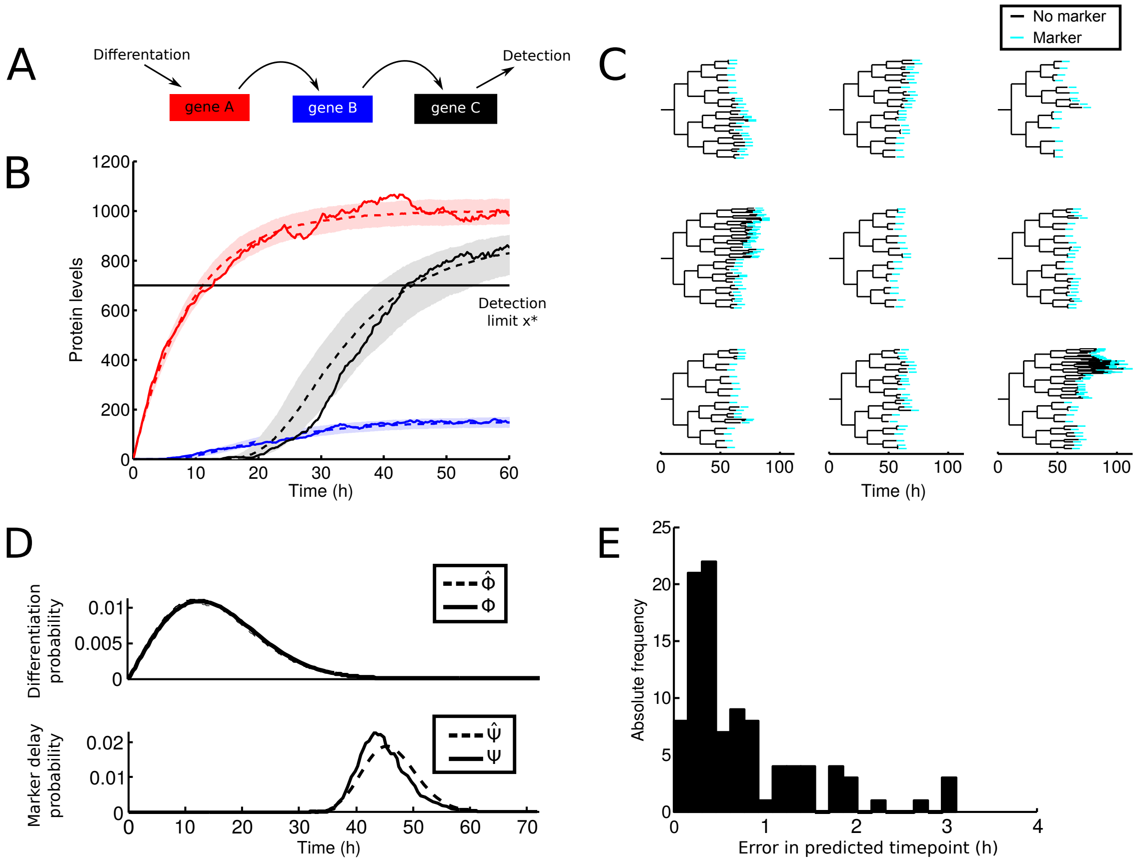

Supplementary Figure 10: The inference model can accurately fit delays arising from cascades of genes. A) In a three-gene cascade, the upstream gene A (red) is activated by the lineage choice and activates its downstream target gene B (blue), which then activates the marker gene C (black). B) Dynamics of the cascade depicted in A). A single realization is shown as solid lines and shaded areas represent 5-95% regions across 1000 realizations. Time is relative to the lineage choice at $t = 0$. The detection limit of gene C is indicated as a horizontal black line. C) Nine genealogies simulated from a linear time-dependent differentiation process (parameters as in Supplementary Figure 8) with a delay arising from the three-gene cascade. D) Estimated differentiation and marker delay probability distributions (dashed lines) from 50 simulated trees agree well with the true distributions (solid lines). The true delay distribution is calculated from 1000 stochastic simulations. E) Histogram of the error in the predicted timepoint of lineage choice. All predicted timepoints in the 100 genealogies from D) are within three hours of the true lineage choice timepoint.

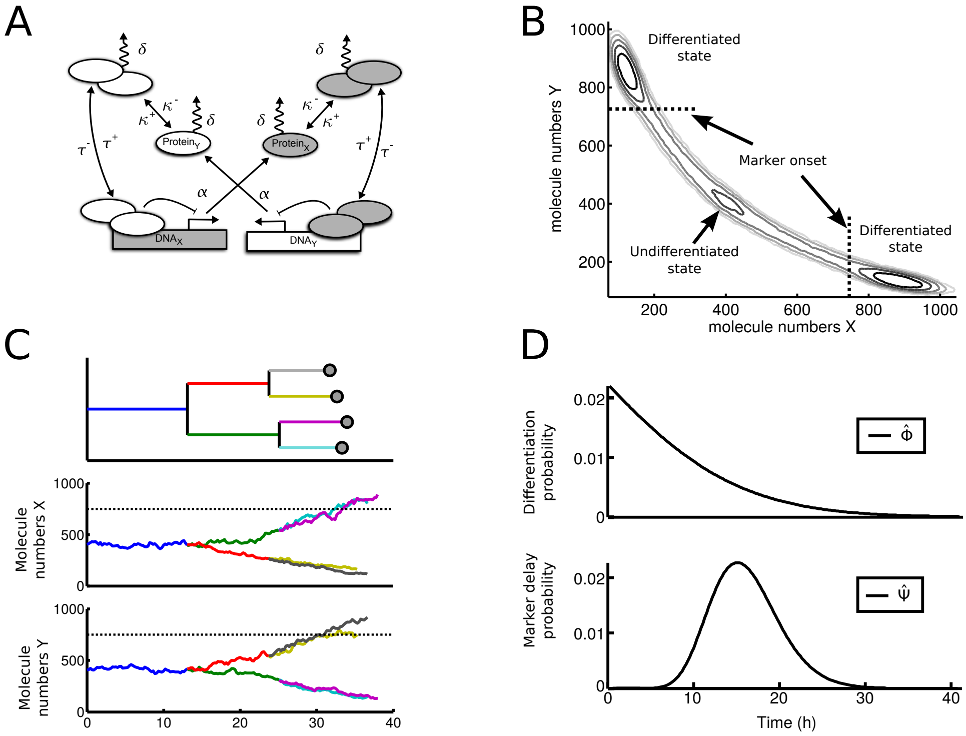

Supplementary Figure 11: Inferring the lineage choice in lineage trees with an underlying toggle switch model recovers the change in the unobserved underlying dynamics. A) A symmetric model of two mutually inhibiting transcription factors X and Y. Transcription and translation are lumped together and mutual inhibition is incorporated in the Hill-type synthesis rates f. Proteins decay with rate γ. B) The model in A) gives rise to one undifferentiated (central) and two differentiated states (upper left, lower right) that can be identified as wells in the quasi-potential $(-\log(P))$ of the system. We define a cell to be differentiated and hence marker positive once it enters the basin of attraction of a differentiated state (gray lines) C) Genealogies are generated from the toggle switch model. Shown are the resulting genealogy (top panel) as well as the timecourses of both factors X and Y (middle panel and lower panel). Individual cells are color coded across panels. D) Estimated differentiation and marker delay probability distributions from 100 simulated observed trees. The differentiation is close to an exponential and the mean marker delay is 15h.

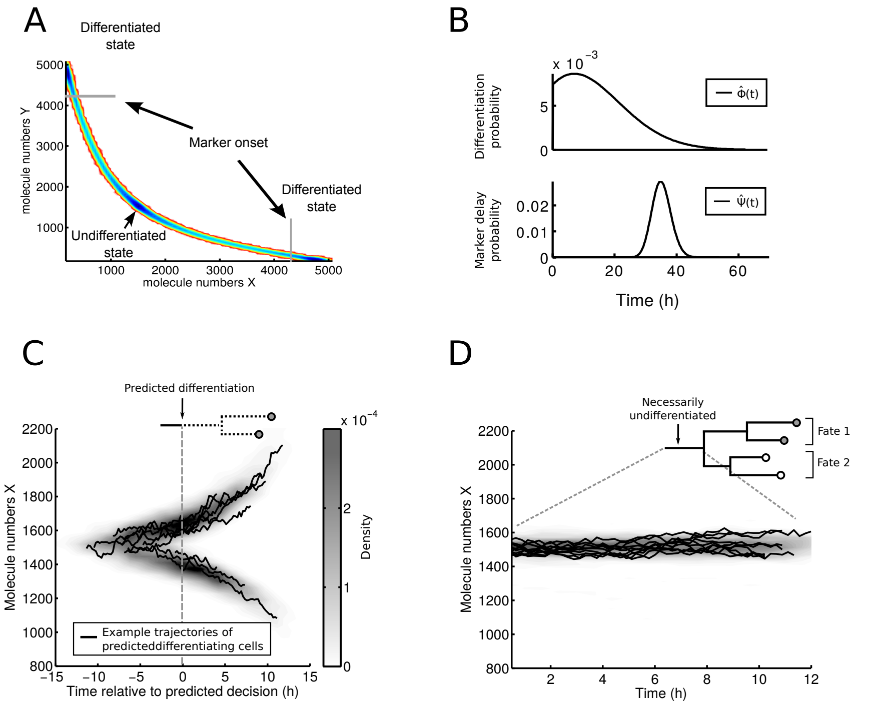

Supplementary Figure 12: Inferring the timepoint of lineage choice in genealogies simulated with a toggle switch model (see Supplementary Figure 11 and main text) and a different set of parameters. A) The model gives rise to a similar attractor landscape as before (compare to Supplementary Figure 11B). However, overall protein numbers are increased. B) Estimated differentiation and marker delay probability distributions from 100 simulated observed trees. C) Trajectories of predicted cells are centered on the timepoint of lineage choice ($t = 0$). D) Trajectories of cells whose progeny differentiates into both fates (see inset). Parameters used for simulation are $\gamma_X = \gamma_Y = 0.29$ h^{-1} (degradation rate), $\alpha_X = \alpha_Y = 1440$ h^{-1} (maximal synthesis rate), $n = 2$ (cooperativity), and $K_X = K_Y = 1000$ (dissociation constants), $x^* = 4500$ (detection threshold).

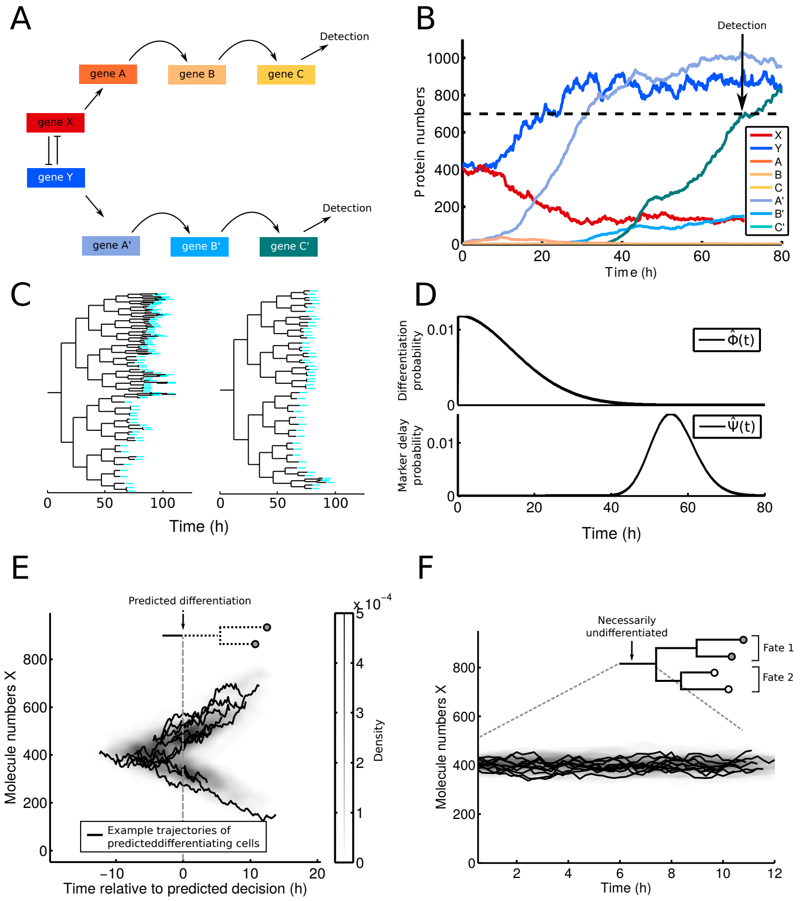

Supplementary Figure 13: A toggle switch coupled to a three-gene cascade. A) Schematic of the model: A toggle switch (genes X and Y, see Supplementary Figure 11) implements the lineage decision while the marker proteins that are detectable (genes C, C') are coupled to the toggle switch via a cascade of intermediate genes. For simplicity, the system is considered to be symmetric, i.e. both cascades share the same parameters. Parameters of the toggle switch and the cascade are the same as for Supplementary Figure 10,11, respectively. B) An exemplary trajectory of the system in A (for simplicity no cell division/branching is considered). The switch tilts at $t = 10$ hours in favor of protein Y, but the outcome of the decision is visible only at $t = 70$ when marker protein C' crosses the detection threshold (dashed black line). C) Two exemplary genealogies simulated with the model in A,B. Detection of the marker proteins C,C' is indicated in cyan. D) Estimated differentiation and marker delay probability distributions from 100 simulated observed trees. E) Trajectories of predicted cells are centered on the timepoint of lineage choice ($t = 0$). F) Trajectories of cells whose progeny differentiates into both fates (see inset).

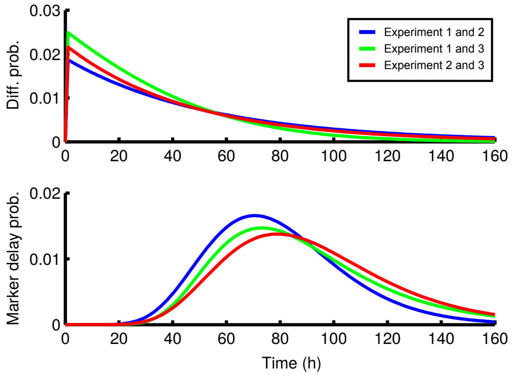

Supplementary Figure 14: Estimating the differentiation and delay distributions across subsampled datasets yields comparable results.

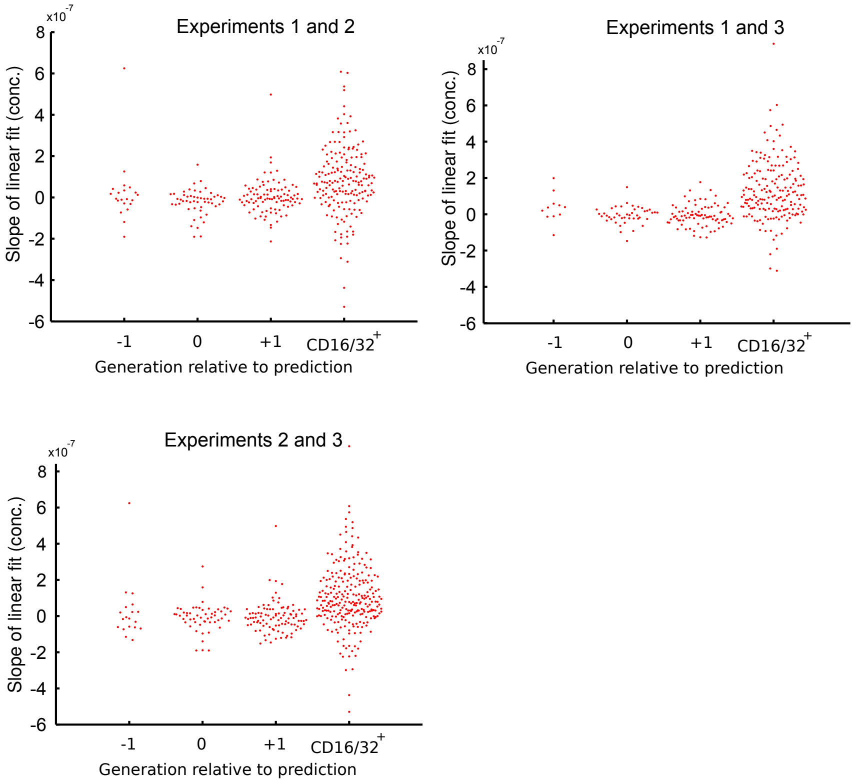

Supplementary Figure 15: PU.1 production does not change in the cells predicted to do lineage choice across the three subsampled datasets.

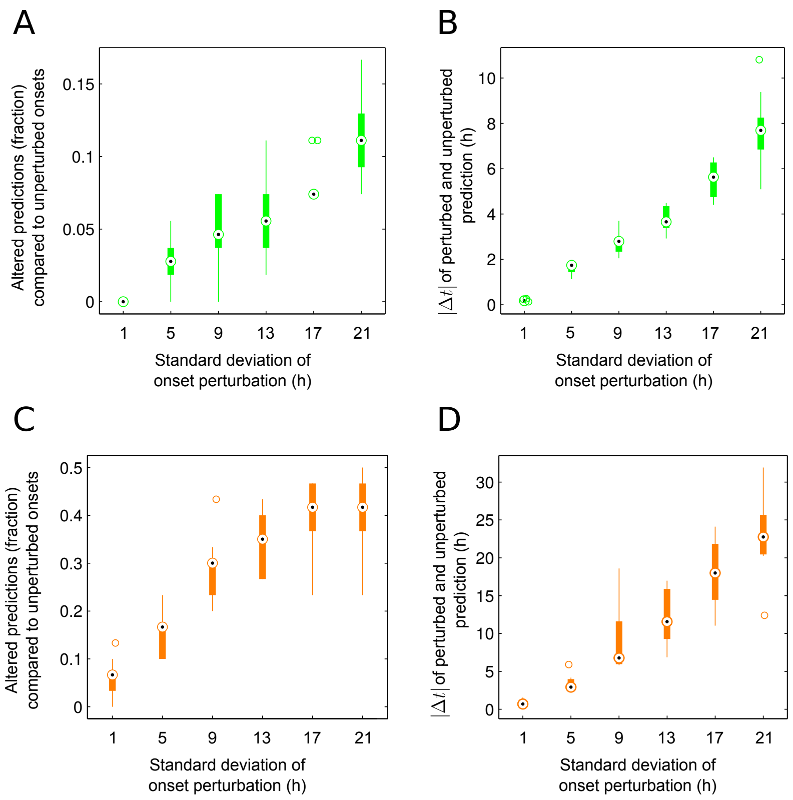

Supplementary Figure 16: Robustness to noise in onset annotation. A,C) Percentage of GM-genealogies (A) and MegE-genealogies (C) where perturbations altered the predicted most likely hidden tree as a function of perturbation strength σ. B,D) Absolute value of the time difference between a predicted lineage choice in unperturbed and perturbed genealogies as a function of perturbation strength σ.

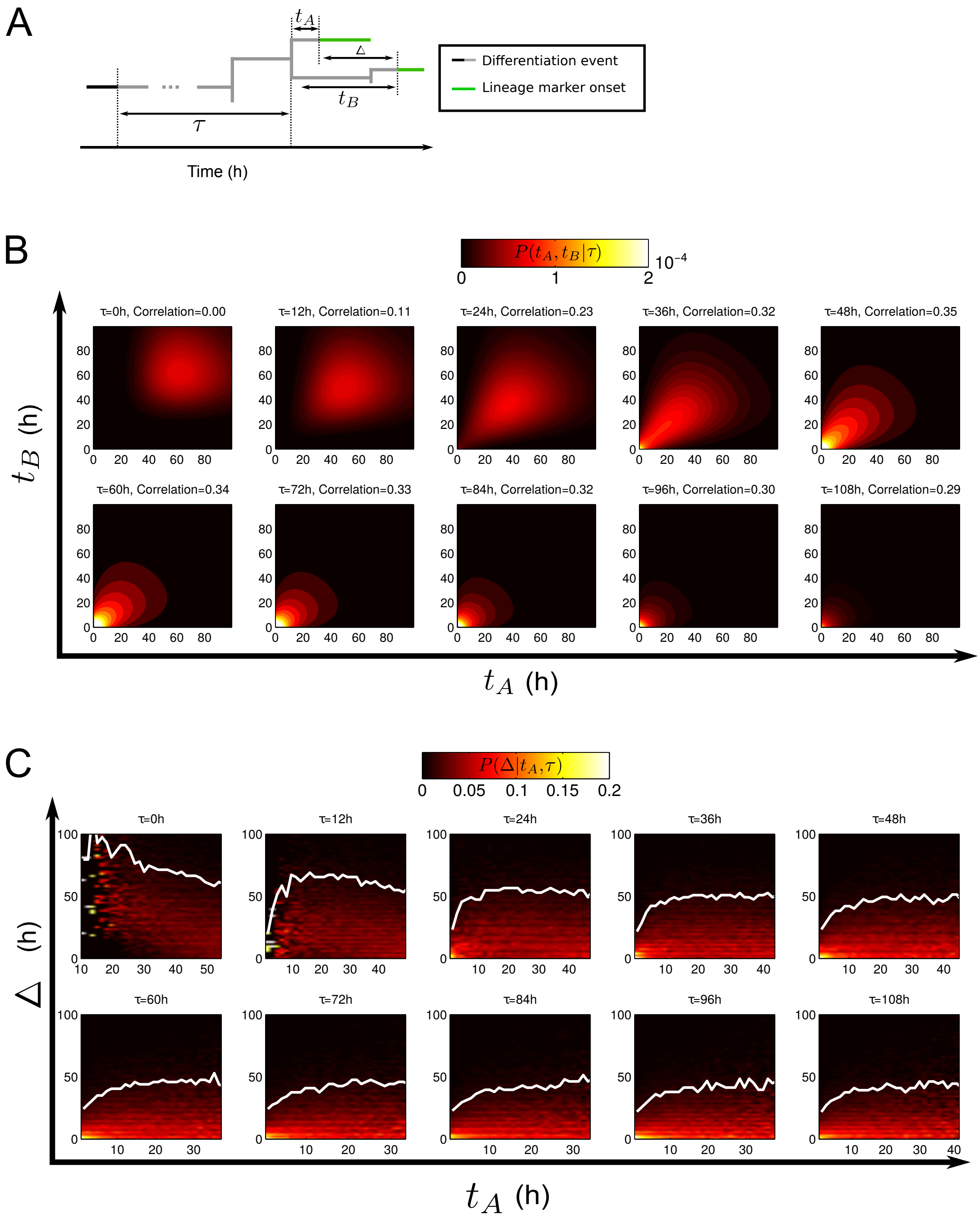

Supplementary Figure 17: Correlations of marker onsets due to the delay process. A) Correlations between onsets are analyzed in terms of i) the time from lineage choice to last common ancestor (τ), ii) time from last common ancestor to the marker onsets (t_A, t_B) and iii) their time difference (Δ). B) The joint distribution $P(t_A, t_B|\tau)$ of onset times for different τ. $\tau = 0$ corresponds to unrelated cells and increasing τ corresponds to increasing shared history of the two onset cells. C) The distribution of onset time difference $P(\Delta|t_A, \tau)$ as a function of shared history (τ) and time of first onset (t_A). The white line marks the 90% quantile of the distributions. If a marker onset in one cell occurs shortly after the two cells split (small t_A), the second cell will most likely have a marker onset in a narrow time window (small Δ).

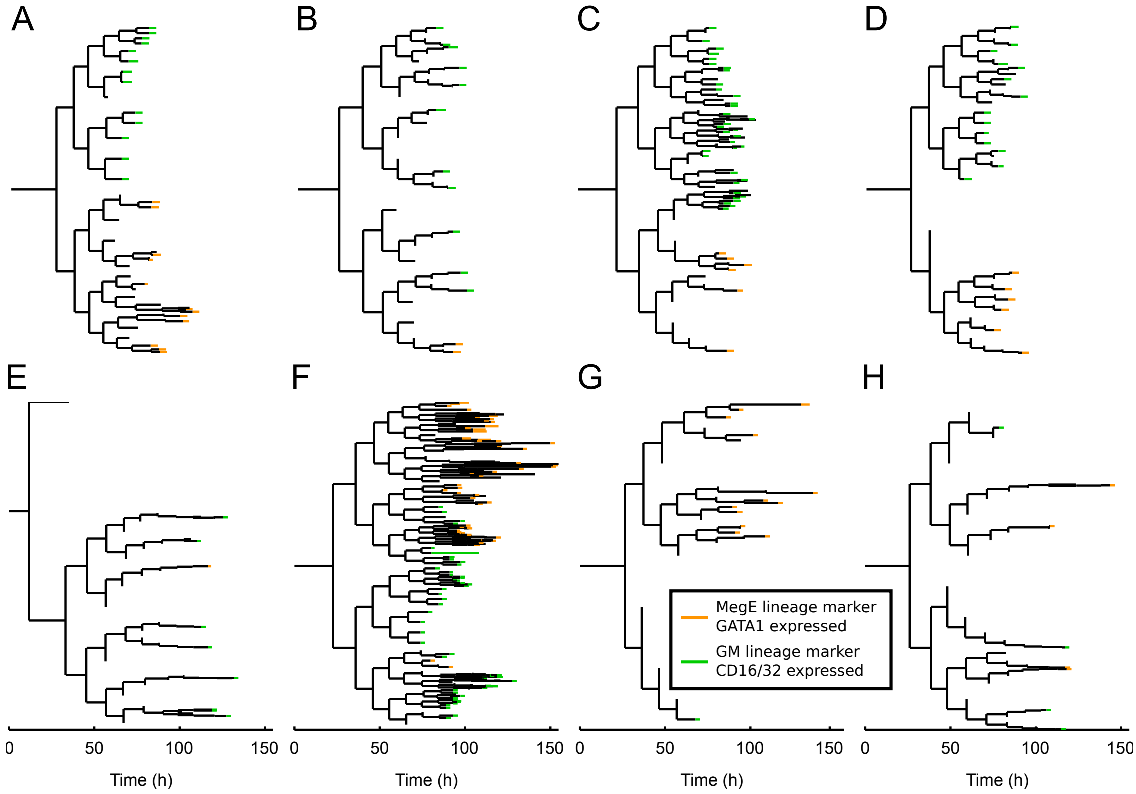

Supplementary Figure 18: Examples of tracked GMMegE genealogies

Supplementary Methods

1 Model

We propose a simple model of lineage choice and delayed readout (see main text, Materials and Methods).

1.1 Differentiation process

Assume that a cell is born at time t_0 and divides at time t_1 and that the differentiation rate is $\lambda(t)$. What is the probability that this cell does not differentiate within its lifetime $[t_0, t_1]$? In general, the number of events k in $[t_0, t_1]$ follows a Poisson distribution

$$P(k|\mu) = \frac{\mu^k}{k!} \cdot \exp(-\mu)$$

with parameter

$$\mu = \int_{t_0}^{t_1} \lambda(t) dt \; .$$

We are interested in the case of no event occurring, hence:

$$P(k = 0|\mu) = \exp(-\mu) = \exp\left(-\int_{t_0}^{t_1} \lambda(t) dt\right) \; .$$

Similarly, the probability $\Phi(t)$ of that cell to differentiate exactly at time $t \in [t_0, t_1]$ is the product of the probability to not differentiate up to time t and the instantaneous differentiation probability $\lambda(t)$, i.e.

$$\Phi(t) = \lambda(t) \exp\left(-\int_{t_0}^{t} \lambda(\tau) d\tau\right) \; . \tag{1}$$

1.2 Marker protein dynamics

The marker protein dynamics of the simplified gene expression system is modeled by the two reactions (see section 2.1.2 in the main paper)

$$\emptyset \xrightarrow{\alpha} X$$
$$X \xrightarrow{\gamma} \emptyset \; ,$$

where the first reaction creates new proteins with rate α and the second reaction degrades proteins with rate γ. The Chemical Master Equation describes how the distribution of protein numbers x evolves over time [2]:

$$\frac{\partial \mathcal{P}(x,t)}{\partial t} = -(\alpha + \gamma x)\mathcal{P}(x,t) + \alpha \mathcal{P}(x-1,t) + \gamma(x+1)\mathcal{P}(x+1,t). \tag{2}$$

This is a linear differential equation and we can also write it in matrix form:

$$\frac{\partial \vec{\mathcal{P}}(t)}{\partial t} = A \cdot \vec{\mathcal{P}}(t),$$

where we choose an ordering of the vector $\vec{\mathcal{P}}(t)$, so that the i-th component corresponds to $\mathcal{P}(i-1,t)$, e.g. $\vec{\mathcal{P}}_1(t)$ is the probability to have 0 proteins at time t. According to this ordering, the matrix A is defined as:

$$A_{ij} = \begin{cases} -(\alpha + \gamma \cdot (i-1)) & \text{if } i = j \\ \gamma \cdot i & \text{if } i+1 = j \\ \alpha & \text{if } i = j+1 \\ 0 & \text{otherwise} \end{cases}$$

We are only interested in the dynamics of the system until the protein numbers exceed the detection threshold x^*. We apply the finite state projection [3] to the master equation, truncating the statespace at state $x^* - 1$ and introducing the absorbing state x^* (see Supplementary Figure 5). This is readily achieved by modifying the matrix A such that

$$A_{x^*,x^*} = 1$$
$$A_{x^*,x^*-1} = 0 \ ,$$

or, equivalently, by extending Eq. 2 with separate equations for the states x^* and $x^* - 1$:

$$\frac{\partial \mathcal{P}(x^*-1,t)}{\partial t} = \alpha \cdot \mathcal{P}(x^*-2,t) - [\alpha + \gamma(x^*-1)] \cdot \mathcal{P}(x^*-1,t)$$
$$\frac{\partial \mathcal{P}(x^*,t)}{\partial t} = \alpha \cdot \mathcal{P}(x^*-1,t) \ .$$

Hence, all probability that leaves the truncated statespace will be collected in the absorbing state x^*.

The first passage time distribution $\Psi_{x_0}(t)$, that is, the probability that the protein number crosses the threshold x^* for the first time at time t starting in state x_0 equals the probability flow from state $x^* - 1$ to x^* at time t [4]:

$$\Psi_{x_0}(t) = \frac{\partial \mathcal{P}(x^*,t)}{\partial t} = \alpha \cdot \mathcal{P}(x^*-1,t|x_0). \tag{3}$$

To obtain $\Psi_{x_0}(t)$ we have to solve the Chemical Master Equation (Eq. 2) up to time t with initial condition $\mathcal{P}(x,0) = \delta_{x,x_0}$. For this simple stochastic model, an analytic expression for the first passage time distribution can be derived in terms of a renewal equation (see supplement of [5]). However, when dealing with tree structures (see Methods section of the main paper), we have to solve the master equation numerically using standard ODE solvers to also obtain the propagator $P_{x \to x'}(t)$, that is the probability to start at state x and after time t arrive at state x', which is not analytically available for a truncated statespace.

2 Inference

2.1 Derivation of the model likelihood

Our goal is to estimate the parameters $(\lambda(t), \alpha, \gamma, x^*)$ of the model (Eq. 1-3) from observed lineage trees in order to predict the lineage choice in a given tree. To that end, we derive the likelihood $\mathcal{L}$ of an observed tree T given the parameters $(\lambda(t), \alpha, \gamma, x^*)$, which is then optimized to find the maximum likelihood estimates.

The likelihood of the observed tree T given parameters $(\lambda(t), \alpha, \gamma, x^*)$ is the sum of likelihoods of the hidden trees $H \in \mathcal{H}(T)$, because these are competing alternatives:

$$\mathcal{L}(T|\lambda(t), \alpha, \gamma, x^*) = \sum_{H \in \mathcal{H}(T)} \mathcal{L}(H|\lambda(t), \alpha, \gamma, x^*) \ . \tag{4}$$

Here, we derive the likelihood $\mathcal{L}(H|\lambda(t), \alpha, \gamma, x^*)$ of a single hidden tree H. We partition the hidden tree into the subtrees D_i induced by the differentiating cells, and a single tree U that contains only undifferentiated cells (see Fig. 2D of the main paper). Due to the Markov property, the likelihood $\mathcal{L}(H|\lambda(t), \alpha, \gamma, x^*)$ factorizes:

$$\mathcal{L}(H|\lambda(t), \alpha, \gamma, x^*) = \mathcal{L}(U|\lambda(t)) \cdot \prod_i \mathcal{L}(D_i|\lambda(t), \alpha, \gamma, x^*) \ . \tag{5}$$

Note that $\lambda(t)$ also appears in the likelihoods for D_i as the root of these subtrees is still undifferentiated for some unknown time (Fig. 2D of the main paper). The first term is readily computed

as the process generating it has no memory and therefore:

$$\mathcal{L}(U|\lambda(t)) = \prod_{c\in U} \mathcal{L}(c|\lambda(t)) \;, \tag{6}$$

$$\mathcal{L}(c|\lambda(t)) = \exp\left(-\int_{t_0^{(c)}}^{t_1^{(c)}} \lambda(t)dt\right) \tag{7}$$

where c is a cell within U that is born at time $t_0^{(c)}$ and divides at time $t_1^{(c)}$ and $\mathcal{L}(c|\lambda(t))$ denotes the likelihood that cell c does not differentiate in its lifetime $[\varsigma_c, \upsilon_c]$ (see section 1.1).

The terms $\mathcal{L}(D_i|\lambda(t), \alpha, \gamma, x^*)$ in Eq. 5 are more difficult to obtain, as the delay process has memory and hence the individual cells of the subtree cannot be treated independently. Also, one has to account for the unknown time interval where the root of the subtree is still undifferentiated. However, these terms can be calculated efficiently using factor graphs (see section 2.2)

Putting together Eq. 4 - 6, we find the likelihood of an observed tree T to be:

$$\mathcal{L}(T|\lambda(t), \alpha, \gamma, x^*) = \sum_{H\in\mathcal{H}(T)} \left[\left(\prod_{c\in U_H} \mathcal{L}(c|\lambda(t))\right) \cdot \prod_i \mathcal{L}(D_i|\lambda(t), \alpha, \gamma, x^*)\right] \;. \tag{8}$$

The sum over H in Eq. 8 consists of a large number of terms (it is double exponential in the number of cells [6]), hence an explicit summation is prohibitive for larger trees. However the sum can efficiently be evaluated using dynamic programming (see section 2.3).

2.2 Factor graph representation of $\mathcal{L}(D_i|\lambda(t), \alpha, \gamma, x^*)$

To obtain the likelihood $\mathcal{L}(D_i|\lambda(t), \alpha, \gamma, x^*)$, we represent each tree D_i as a factor graph (Supplementary Figure 6) and perform inference via message passing [7].

For each non-root cell c in D_i, we create two nodes, one representing the state $X_c \in \{0, 1, \ldots, x^*\}$ of the cell c at its first timepoint, the other representing its state $Y_c \in \{0, 1, \ldots, x^*\}$ before division (Supplementary Figure 6). One additional node τ_c per cell represents the lifetime of that cell (black circles in Supplementary Figure 6). These three nodes associated with cell c are linked via the factor f_c (squares in Supplementary Figure 6), which expresses the probability to reach state Y_c in time τ_c starting from state X_c. The factor f_c is thus the propagator of the associated Markov process: $f_c(X_c, Y_c, \tau_c) = P_{X_c\to Y_c}(\tau_c)$. It is obtained by solving the Chemical Master Equation (Eq. 2) numerically. Individual cells are linked via cell division factors g_c (diamonds in Supplementary Figure 6) that couple the last state of the mother (Y_c) to the first states (X_{d_1}, X_{d_2}) of the two daughters d_1, d_2. For simplicity, we neglect partitioning of molecules at cell division and assume that this factor is the identity: $g_c(Y_c, X_{d_1}, X_{d_2}) = \delta_{Y_c,X_{d_1}} \cdot \delta_{Y_c,X_{d_2}}$. This ensures that both subtrees inherit the same state from the mother cell. This can be extended by binomial partitioning of molecules [8], e.g. via defining the factor g_m relating the number of proteins in the mother cell m and its two daughter cells d_1, d_2 as

$$g_m(Y_m, X_{d_1}, X_{d_2}) = \underbrace{\binom{Y_m}{X_{d_1}} p^{X_{d_1}}(1-p)^{Y_m - X_{d_1}}}_{\text{probability of split}} \cdot \underbrace{\delta_{X_{d_1}+X_{d_2},Y_m}}_{\text{mass conservation}} \;.$$

Finally, the root cell r is represented by node Y_r, representing the state before division and is coupled to a factor f_r implementing the integration over the unknown timepoint of differentiation within r:

$$f_r(Y_r) = \int_{t_0^{(r)}}^{t_1^{(r)}} \Phi(t|\lambda(t)) P_{0\to Y_r}(t_1^{(r)} - t)dt \;. \tag{9}$$

The first term in the integral gives the probability that the differentiation occurred at time t and the second term is the probability to reach protein number Y_r in the remaining cell cycle starting from zero proteins.

In this factor graph, which compactly describes the joint distribution over all variables

$$P(X_1, \ldots, X_n, Y_1, \ldots, Y_n, \tau_1, \ldots, \tau_n) ,$$

certain nodes are observed (filled nodes in Supplementary Figure 6): We observe the cell cycle length τ_i of all cells and we also know the protein number of the leave cells l, because we observe the marker in these cells: $Y_l = x^*$. The desired likelihood $\mathcal{L}(D_i|\lambda(t), \alpha, \gamma, x^*)$ of subtree D_i is equal to the evidence of the data, $\mathcal{L}(D_i|\lambda(t), \alpha, \gamma, x^*) = p(Y_{l_1} = x^*, \ldots, Y_{l_L} = x^*)$, i.e. one hast to integrate the joint distribution over all unobserved nodes. The evidence is calculated with the sum-product algorithm on this factor graph [7] (see section 2.2.1).

2.2.1 Message Passing

To evaluate the likelihood $\mathcal{L}(D_i|\lambda(t), \alpha, \gamma, x^*)$ of the subtrees D_i, we perform message passing on the factor graph. Using the notation of [7], we define messages $\mu_{X\to f}$ going from node X to factor f as

$$\mu_{X\to f}(x) = \prod_{l\in \mathrm{ne}(X)\setminus f} \mu_{l\to X}(x) \tag{10}$$

The factors l are all factors connected to node X except factor f (ne(x) denotes neighbors in the graph). Messages going from a factor f to a node X are defined as

$$\mu_{f\to X}(x) = \sum_{x_1} \ldots \sum_{x_M} f(x, x_1, \ldots, x_M) \prod_{Y\in \mathrm{ne}(f)\setminus X} \mu_{Y\to f}(y) , \tag{11}$$

where $x_1, \ldots, x_M$ are nodes connected to factor f in addition to x and Y denotes all nodes that are connected to factor f except X. For a detailed derivation of these expressions, we refer the interested reader to Chapter 8.4 of [7].

In the following, we give a brief example of the message passing performed on the graphical model shown in Supplementary Figure 6A, where marker onset is observed in cells 4 and 5. Note that for readability, we ignore the lifetime nodes (black circles in Supplementary Figure 6B) in the message passing scheme and simply treat them as constants in the associated factors. Also we omit the dependence of the factors on the parameters $(\lambda(t), \alpha, \gamma, x^*)$.

- We start by sending a message from Y_4 to f_4. Because Y_4 has no incoming messages, $\mu_{Y_4\to f_4}(y_4) = 1$, but as Y_4 is observed (marker onset at x^*), its state is made definite via the δ-function.
$$\mu_{Y_4\to f_4}(y_4) = \delta_{y_4,x^*} .$$
- Next, we send a message from f_4 to X_4 using Eq. 11:
$$\begin{aligned}\mu_{f_4\to X_4}(x_4) &= \sum_{y_4} f_4(x_4, y_4) \prod_{m\in \mathrm{ne}(f_4)\setminus x_4} \mu_{m\to f_4}(m) \\ &= \sum_{y_4} f_4(x_4, y_4) \mu_{Y_4\to f_4}(y_4) \\ &= \sum_{y_4} P_{x_4\to y_4}(\tau_4) \mu_{Y_4\to f_4}(y_4) = \sum_{y_4} P_{x_4\to y_4}(\tau_4) \cdot \delta_{y_4,x^*} = P_{x_4\to x^*}(\tau_4) ,\end{aligned}$$
where in the last line, we used the definition of the factor f_4 as the propagator of the Markov process. The same procedure is applied to obtain the message $\mu_{f_5\to X_5}(x_5)$.

- In the next step, messages $\mu_{X_4\to g_2}(x_4)$ and $\mu_{X_5\to g_2}(x_5)$ are sent to the cell division factor g_2. From the definition in Eq. 10, we see that the nodes X_4, X_5 simply pass through the incoming messages unchanged:

$$\mu_{X_4\to g_2}(x_4) = \mu_{f_4\to X_4}(x_4) = P_{x_4\to x^*}(\tau_4)$$
$$\mu_{X_5\to g_2}(x_5) = \mu_{f_5\to X_5}(x_5) = P_{x_5\to x^*}(\tau_5)$$

- Again applying Eq. 11, we find that the cell division factor g_2 sends to Y_2 the message

$$\begin{aligned}\mu_{g_2\to Y_2}(y_2) &= \sum_{x_4}\sum_{x_5} g_2(y_2, x_4, x_5)\cdot \mu_{X_4\to g_2}(x_4)\cdot \mu_{X_5\to g_2}(x_5)\\ &= \sum_{x_4}\sum_{x_5} \delta_{y_2,x_4}\delta_{y_2,x_5}\cdot \mu_{X_4\to g_2}(x_4)\cdot \mu_{X_5\to g_2}(x_5)\\ &= \mu_{X_4\to g_2}(y_2)\cdot \mu_{X_5\to g_2}(y_2)\\ &= P_{x_2\to x^*}(\tau_4)\cdot P_{x_2\to x^*}(\tau_5)\ .\end{aligned}$$

Here, we used the assumption of perfect state inheritance from mother to daughter as described before.

- Finally, a message has to be sent from the integration factor f_2 towards Y_2:

$$\begin{aligned}\mu_{f_2\to Y_2}(y_2) &= f_2(y_2)\\ &= \int_{t_0^{(2)}}^{t_1^{(2)}} dt'\ \Phi(t|\lambda(t))P_{0\to y_2}(t_1^{(2)} - t)\ .\end{aligned}$$

- To obtain the likelihood of the whole subtree, we have to multiply all messages arriving at Y_2 and sum over all states y_2:

$$\mathcal{L}(D_2) = \sum_{y_2} \mu_{f_2\to Y_2}(y_2)\cdot \mu_{g_2\to Y_2}(y_2)\ .$$

2.3 Resolving the combinatoric complexity

In section 2.1, Eq. 8, we derived the likelihood of one observed tree T given the model parameters $(\lambda(t), \alpha, \gamma, x^*)$ as the sum over all hidden trees $\mathcal{H}(T)$:

$$\mathcal{L}(T|\lambda(t), \alpha, \gamma, x^*) = \sum_{H\in\mathcal{H}(T)} \left[\left(\prod_{c\in U_H} \mathcal{L}(c|\lambda(t))\right)\cdot \prod_{D_i\in D_H} \mathcal{L}(D_i|\lambda(t), \alpha, \gamma, x^*)\right]\ .$$

The sum over $\mathcal{H}(T)$ contains a potentially large number of terms (the size of $\mathcal{H}(T)$ it is doubly exponential in the number of generations [6], or equivalently, it is exponential in the number of cells). For a full binary tree with n generations, the number of hidden trees is equivalent to the number y_n of strongly binary trees (rooted trees whose nodes have either zero or two children) with generations $\leq n$. This number is defined by the quadratic map $y_n = y_{n-1}^2 + 1$ with $y_0 = 1$. For example, for a tree with five generations this evaluates to $y_5 = 458330$ hidden trees.

Hence, carrying out the sum explicitly is prohibitive for large trees. We thus devised a dynamic programming approach that avoids the explicit enumeration of hidden trees H. First we introduce two convenient abbreviations:

$$S(i) = \mathcal{L}(D_i|\lambda(t), \alpha, \gamma, x^*)$$

is the probability of the subtree rooted in cell i, which we obtain by inference on the factor graph (see section 2.2.1). Furthermore, we define

$$\tilde{P}(i) = \prod_{c\in LA(i)} \mathcal{L}(c|\lambda(t))\ .$$

Algorithm 1: Recursive algorithm to calculate $LA(i)$. We assume an arbitrary but fixed ordering of the tree, i.e. we can rely on the definition of a "left" child cell.

```
Input: Cell i, tree T
Output: Set of ancestor cells LA(i)
Algorithm LA(i,T)
    if i ∉ T then
        return ∅ ;                              // if cell does not exist in T
    else if cell i has a left sibling then
        return {i} ;   // as all cells above c are contained in LA(left sibling)
    else
        m = mother(i) ;                                  // its direct ancestor
        return {c, LA(m,T)} ;                // continue recursion in mother cell
    end
```

Here, $LA(i)$ is defined via Algorithm 1 and denotes a particular set of undifferentiated ancestor cells, such that every undifferentiated cell in the tree is contained in only one set $LA(i)$. $\mathcal{L}(c|\lambda(t))$ denotes the probability of cell c being undifferentiated (see Eq. 7).

Now we define $\kappa(i)$, which aids us in calculating the value of the overall sum in Eq. 8:

$$\kappa(i) = \begin{cases} S(i) \cdot \tilde{P}(i) & \text{if } i \in L \\ S(i) \cdot \tilde{P}(i) + \kappa(v) \cdot \kappa(w) & \text{if } i \notin L, \end{cases} \tag{12}$$

where L denotes the set of leaves of T and v, w are daughters of cell i. If cell i is a leave, the probability is just the product of the subtree probability and the probability to be still undifferentiated (corrected for the multiple counting, hence $\tilde{P}$). If cell i is not a leave, its contribution to the combinatorics is the following: Either it differentiates and generates the subtree below (first term) or it just leads to the combinations of the two subtrees (starting at cells v, w) below (resulting in all possible combinations of the events in the subtree). Finally, we're only interested in

$$\mathcal{L}(T|\lambda(t), \alpha, \gamma, x^*) = \kappa(1) \tag{13}$$

and we can use the recursive rule Eq. 12 to calculate the likelihood of an observed tree (Eq. 8) efficiently.

2.4 Finding the most likely hidden tree

Given an observed tree T and a set of parameters $(\lambda(t), \alpha, \gamma, x^*)$ the most likely hidden tree $\hat{H}$ can be determined according to Eq. 5 of the main text:

$$\hat{H} = \underset{H \in \mathcal{H}(T)}{\operatorname{argmax}} \mathcal{L}(H|\lambda(t), \alpha, \gamma, x^*) \ .$$

This can be done efficiently without explicitly enumerating the entire set $\mathcal{H}(T)$, but using the quantities $\kappa(i), S(i)$ defined in section 2.3. The key idea is that $\kappa(i)$ represents an upper bound on the contribution of the subtree rooted in i (where cell i or any set of descendants of i differentiates) to the likelihood. If the currently best solution H^* already has larger likelihood than $\kappa(i)$, all solutions with differentiation events downstream of cell i can be removed from the search space. Pseudocode is shown in Algorithm 2, which yields

$$\hat{H} = \text{enumerate}(r, 0)$$

where r is the root of T.

A similar algorithm can be used to obtain the n-most likely trees $H \in \mathcal{H}$ and their corresponding likelihoods $\mathcal{L}(H|\lambda(t), \alpha, \gamma, x^*)$. In Supplementary Figure. 7 the 100 most likely hidden trees of the first lineage tree shown in Supplementary Figure. 2A are enumerated.

Algorithm 2: Recursive algorithm to calculate $\text{argmax}_{H\in\mathcal{H}(T)}\,\mathcal{L}(H|\lambda(t),\alpha,\gamma,x^*)$

```
Input: Cell c, tree T, κ, S, currently best probability p*
Output: Set of differentiating cells C, probability p
Algorithm enumerate(c, p*)
  if c ∈ Leaves(T) ;                                          // base case
   then
    if S(c) < p* then
      // worse than current best solution
      return C = ∅, p = p*
    else
      return C = {c}, p = S(c)
    end
  else
    upperBound = κ(c) − S(c);                                  // recursion
    if upperBound < p* then
      return C = ∅, p = p*
    else
      // recursion into the daughter cells d1, d2 of c
      c_l, p_l = enumerate(d1, p*)
      c_r, p_r = enumerate(d2, p*)
      if p_l · p_r < S(c) then
        // combining the subtrees is worse than taking c
        return C = {c}, p = S(c)
      else
        // combine the subtrees
        return C = c_l ∪ c_r, p = p_l · p_r
      end
    end
  end
```

2.5 Accounting for lineage choice before movie start

Due to sorting impurity, it is possible that cells entering the time-lapse microscopy experiment at t_0 are already committed towards one or the other lineage. While mature cells that already express lineage markers at t_0 can easily be removed (these cells are usually not even tracked), we still must account for cells that made their lineage choice at some time $t < t_0$ but not show the lineage markers due to delay. It is straightforward to include this into our model by modifying the likelihood function Eq. 4 as:

$$\mathcal{L}(T|\lambda(t),\alpha,\gamma,x^*,\pi) = \pi\cdot\mathcal{L}(H_0|\lambda(t),\alpha,\gamma,x^*) + (1-\pi)\sum_{H\in\mathcal{H}(T)}\mathcal{L}(H|\lambda(t),\alpha,\gamma,x^*)\ , \tag{14}$$

where $\pi\in[0,1]$ is the fraction of genealogies that decide their lineage before t_0 and H_0 is the hidden tree corresponding to an already differentiated cell entering the experiment. π is estimated with the other parameters of our model $\lambda(t),\alpha,\gamma,x^*$. To calculate $\mathcal{L}(H_0|\lambda(t),\alpha,\gamma,x^*)$, notice that there is no need to decompose the hidden tree into undifferentiated and differentiated subtrees. One can directly translate H_0 into a factor graph as described in section 2.2. One only has to modify the expression of the integration factor f_r (compare to Eq. 9):

$$f_r(Y_r) = \sum_{x=0}^{x^*-1} P(x)\cdot P_{x\to Y_r}(t_1^{(r)}-t_0^{(r)})\ , \tag{15}$$

where $P(x)=\frac{1}{x^*}$ is a uniform prior distribution over the protein expression state when the cell enters the experiment. Here, we account for the fact that a cell might enter the experiment at

various stages of the delay and integrate over this unknown quantity.

Supplementary Note 1: Inference on synthetic data

We first test our method on synthetic data, choosing parameters θ and η, which give rise to a particular differentiation and delay distribution (see Supplementary Figure 8A, gray). We generate 50 lineage trees according to those distributions, where we do not observe the timepoint of lineage choice, but only the marker onset (Supplementary Figure 8B). By solving the optimization problem in Eq. 4 for our dataset, we obtain a maximum likelihood estimate $(\hat{\theta}, \hat{\eta})$ and compute the corresponding differentiation and delay distributions ($\hat{\Phi}$, $\hat{\Psi}$ in Supplementary Figure 8A, solid lines), which closely match the data generating distributions(Φ, Ψ, grey). Furthermore, we predict the most likely hidden trees via Eq. 5 and calculated the distance in time between true and predicted timepoints of lineage choice (Supplementary Figure 8C). The difference in predicted and true timepoint of decision is 1.35 h on average. Thus, our method can accurately recover the underlying parameters and faithfully predict the true timepoint of lineage choice just from observed marker correlations.

We now test the predictions of our model trained with the 50 genealogies on a independent test set of 100 genealogies. For each of the 100 trees in the test set, we obtain its most likely hidden tree via Eq. 5, thereby predicting the timepoint of lineage choice. Comparing these predictions to the ground truth, we find that for 77 of 100 observed trees, we predict the correct hidden tree. In terms of single cells, we correctly recall 187 of 229 differentiating cells, while 27 cells are falsely identified as differentiating (for the confusion matrix, see Supplementary Table 1).

To systematically validate the algorithm's performance, we repeat the above analysis for a set of 100 parameters $(\theta, \eta)_i, (i = 1, \ldots, 100)$, randomly sampled from the intervals given in Supplementary Table 2. We simulate 50 trees T_i for each $(\theta, \eta)_i$ and perform the maximum likelihood estimation using Eq. 4 (main text). For each set of trees T_i, we obtain estimates of the underlying differentiation and delay distributions ($\hat{\Phi}^i$ and $\hat{\Psi}^i$). To measure the agreement between true and estimated distributions (p and $\hat{p}$'), we calculate their L_1-distance as:

$$L_1(p, \hat{p}) = \sum_t |p(t) - \hat{p}(t)| \ .$$

A histogram of the L_1-distances between estimated and true distributions is shown in Supplementary Figure 9. The distances in the differentiation distributions are generally small ($\text{Quantile}_{0.95} = 0.25$, Supplementary Figure 9A). For the delay distributions these distances are sometimes larger ($\text{Quantile}_{0.95} = 0.70$, Supplementary Figure 9B), suggesting that it is more difficult to extract these parameters from the data. Overall, the analysis suggests that we can reconstruct the underlying parameter from the observation with good accuracy.

		Predicted	
		differentiating	not differentiating
Ground truth	differentiating	TP=187	FN=42
	not differentiating	FP=27	TN=792

Supplementary Table 1: Confusion matrix for the predictions of lineage choice in 100 genealogies with linear time-dependent differentiation and a single-gene delay. Training was performed on an independent set of 50 genealogies. (TP: true positive, FN: false negative, FP: false positive, TN: true negative)

	a_0	a_1	α	γ	x^*
lower bound	10^{-15}	10^{-15}	10^{-6}	10^{-15}	1
upper bound	10^{-7}	10^{-7}	10^{-1}	10^{-3}	150

Supplementary Table 2: Parameter ranges used to simulate the synthetic datasets.

Supplementary Note 2: Inference on a cascade of genes

Until now we have used a simple gene expression model combined with a detection limit of marker onset to explain correlations in trees. Here, we assess if our simple model can cope with a more realistic delay process consisting of a cascade of three genes (Supplementary Figure 10A):

$$\emptyset \xrightarrow{\alpha_A} A \xrightarrow{\gamma_A} \emptyset$$
$$\emptyset \xrightarrow{\tilde{\alpha}_B(A)} B \xrightarrow{\gamma_B} \emptyset$$
$$\emptyset \xrightarrow{\tilde{\alpha}_C(B)} C \xrightarrow{\gamma_C} \emptyset\,.$$

Upon lineage choice, expression of the first gene in the cascade (gene A in Supplementary Figure 10A) is triggered, which in turn activates expression of its downstream target (gene B in Supplementary Figure 10A). Gene B in turn activates gene C, whose expression can be detected once crossing a detection limit x^* (as in Supplementary Figure 10B).

Activation is governed by a Hill function, such that the production rate $\tilde{\alpha}_B(A)$ of gene B is an increasing function of the number of activator molecules:

$$\tilde{\alpha}_B(A) = \alpha_B \cdot \frac{K_A^n}{K_A^n + A^n}$$
$$\tilde{\alpha}_C(B) = \alpha_C \cdot \frac{K_B^n}{K_B^n + B^n},$$

with maximal synthesis rates $\alpha_A = \alpha_C = 100\ \mathrm{h}^{-1}, \alpha_B = 22\ \mathrm{h}^{-1}$, cooperativity $n = 5$ and dissociation constants $K_A = 800, K_B = 100$. Degradation rates are set to $\gamma_A = \gamma_B = \gamma_C = 0.1\ \mathrm{h}^{-1}$. The dynamics of this stochastic process leads to a long and heterogeneous delay ranging from 35 to 60 hours after lineage choice (see Supplementary Figure 10D).

We now simulate 50 genealogies from a time-dependent differentiation process (parameters as in Supplementary Figure 8, main text) and the three-gene cascade (a sample of nine genealogies is shown in Supplementary Figure 10C). As before, we fit the model to the data via Eq. 4 (main text). Note that the model still assumes a single gene delay process. Comparing the estimated to the true distributions, we observe good agreement (Supplementary Figure 10D). We note a slight deviation for the delay distribution, which arises due the difference in delay processes (a single gene as opposed to a cascade).

We evaluate the performance of the fitted model on an independent test set of 100 genealogies. For 91 of 100 genealogies, the most likely hidden tree (obtained via Eq. 5, main text) indeed corresponds to the true underlying lineage choice scenario. Performance in terms of single cell prediction is summarized in Supplementary Table 3. Note that due to the long delay many non-differentiating cells are present, which are mostly classified correctly. In terms of time difference between predicted and actual timepoint of lineage choice, we find that the predicted timepoint is always within 3 hours of the true timepoint (Supplementary Figure 10E) and the misclassifications in Supplementary Table 3 happen close to cell division (where e.g the mother cell might differentiate at the end of its cell cycle, but the methods predicts that its daughter cells difference at the beginning of their cell cycles).

		Predicted	
		differentiating	not differentiating
Ground truth	differentiating	TP=180	FN=18
	not differentiating	FP=9	TN=5401

Supplementary Table 3: Confusion matrix for the predictions of differentiating cells in 100 genealogies in scenario of linear time-dependent differentiation and a three-gene cascade delay. Training was performed on an independent set of 50 genealogies. (TP: true positive, FN: false negative, FP: false positive, TN: true negative)

Supplementary Note 3: Inference on a toggle switch model

Next, we test if our simplifying assumptions of independent differentiation and delay due to a stochastic gene expression process are compatible with existing paradigms of lineage choice. To address this, we implement the popular toggle switch model that is thought to drive binary lineage decision [9, 10, 11, 12, 13, 14, 15] composed of two mutually repressing transcription factors X and Y (see Supplementary Figure 11A). The model is summarized by four chemical reactions (synthesis and degradation of the respective proteins):

$$\begin{aligned} \emptyset &\xrightarrow{f(Y)} X \\ X &\xrightarrow{\gamma} \emptyset \\ \emptyset &\xrightarrow{f(X)} Y \\ Y &\xrightarrow{\alpha} \emptyset\,. \end{aligned}$$

Mutual inhibition of both factors is not modeled explicitly as binding and dissociation of the repressor proteins to the promoters, but is incorporated into the propensities of the synthesis reactions (assuming fast binding and dissociation of the repressor):

$$\begin{aligned} f(Y) &= \frac{\alpha}{1+(\frac{Y}{k_d})^n} \\ f(X) &= \frac{\alpha}{1+(\frac{X}{k_d})^n} \end{aligned}$$

In these Hill-type regulatory functions, α is the maximal synthesis rate (when no repressor is present), k_d is the amount of repressor needed to reduce synthesis to 50%, and n is the cooperativity of the repressor protein. For simplicity, we consider a symmetric system, where X and Y share the same set of parameters.

This model exhibits three stable states (see Supplementary Figure 11B): One state, where both proteins are expressed at similar levels is associated with an undifferentiated cell. In the two other states, either one or the other protein is strongly upregulated, thereby repressing the other, representing two mutually exclusive differentiated lineages. Differentiation occurs via noise driven transitions from the undifferentiated to one of the differentiated states. Using Gillespie's algorithm [16] we simulate trees from this toggle switch model (see Supplementary Figure 11C): The root cell of each tree starts in the undifferentiated state, from where it evolves over time according to the laws of the underlying toggle switch model until it divides. The cell cycle time is drawn from a log-normal distribution ($\mu = 12\text{h}, \sigma = 1\text{h}$). Upon division, two identical daughter cells are created that inherit the state of the mother cell (we ignore random partitioning of molecules at cell division for simplicity). Once a cell arrives at one of the differentiated states (defined as crossing $x^* = 750$ molecules of either protein, black dashed lines in Supplementary Figure 11B,C), we annotate this cell as being differentiated (gray circles in Supplementary Figure 11C). We assume that we cannot observe the toggle switch dynamics of X and Y that drives lineage choice. It is apparent that the lineage decision, that is, the time when the system leaves the undifferentiated state, occurs earlier than the observation of the marker (gray circles in Supplementary Figure 11C).

We then apply our inference method and learn the model parameters θ, η from 100 simulated trees. Note that these parameters do not correspond to the parameters of the toggle switch model itself, but to a simplified description of the differentiation and delay process. The estimated differentiation distribution $\hat{\Phi}$ is exponential (Supplementary Figure 11D), indicating that the process of leaving the undifferentiated state is well described by a point process with constant rate. Intuitively, this rate reflects the frequency of a large fluctuation that pushes the cell out of the undifferentiated state. Inspecting the estimated delay distribution $\hat{\Psi}$ we find an average delay of 15 ± 4 hours, which visually coincides with the time between divergence of molecule numbers and annotations that we observe in Supplementary Figure 11C. Next, we study the underlying timecourses of the toggle switch in context of the predicted differentiation decisions. Using Eq. 5

(main text), we predict the differentiating cells and the timepoint of differentiation within those cells. The timecourses of the two proteins X and Y in these predicted cells are then aligned at the predicted differentiation timepoint ($t_d = 0$ on the x-axis of Fig. 4E, main text). This timepoint coincides with the point where trajectories diverge, i.e. the method detects the timepoint where the toggle switch tilts into one or the other direction, corresponding to the lineage choice. To further quantify this, we fit linear models to the timecourse of each single cell, group cells accordingly into generations before, at, and after the predicted differentiation and assess the protein production (slope of the linear fit) of X and Y in these cells (Fig. 4F,G, main text). While the production is zero and protein levels stay constant in generations before the predicted differentiation decision, the production clearly changes in cells that are predicted to differentiate (Fig. 4F,G, main text). For generations after the predicted differentiation decision and at the observed marker onset, production plateaus and decreases as the toggle switch approaches one or the other differentiated state.

We performed similar analyses for a different parameter set (see Supplementary Figure 12) and for a toggle switch coupled to a three-gene marker cascade (see Supplementary Figure 13) and obtained the same result: In the predicted cells the balance of the two toggle switch proteins is broken and the system tilts towards one or the other differentiated state.

Thus, our method is able to reconstruct the timepoint of lineage choice even if the decision model is more complex and implemented via a genetic toggle switch.

Supplementary Note 4: Robustness with respect to experimental data

To test whether the results from the hematopoiesis dataset are robust across the $n = 3$ experiments, we subsampled the dataset, leaving out an entire experiment one at a time. For the resulting three datasets, we performed inference with our model by solving Eq. 4 (main text). The estimated distributions Φ, Ψ are similar across these three runs (Supplementary Figure 14).

Due to the similarly estimated distributions, also the cells predicted to differentiate are mostly the same across the three runs. Most importantly, the slopes of PU.1 show the same behaviour around the predicted time of lineage choice (Supplementary Figure 15).

Supplementary Note 5: Robustness to onset perturbations

Lineage marker onsets (CD16/32 and Gata1) are manually annotated on the genealogies during tracking and independently verified in later processing of the data and are therefore believed to be of high accuracy and confidence. Nevertheless, we investigate how "measurement noise" on these onsets would affect our model. Therefore, we perturbed each annotated onset in the dataset with noise. We added a Gaussian distributed time interval Δt to each onset time t, displacing the onset in time along the tree and creating a new perturbed onset t' as

$$t' = t + \Delta t, \ \Delta t \sim \text{Normal}(0, \sigma^2)] \ ,$$

where σ controls the magnitude of the perturbation. Note that onsets can be shifted into mother or daughter cells. Consistency of onsets was enforced: For two sister cells with onsets, if a perturbation in one sister cell shifts the onset into the mother, the onset in the other sister is removed.

After perturbing every onset once in the dataset, we evaluated how the prediction of lineage choice changed in the genealogies. First, we counted how often the prediction of cells changed from the unperturbed to the perturbed version of our dataset (Supplementary Figure 16A,C). Second, we measured the time interval between predicted lineage choice events in unperturbed genealogies and their corresponding predictions in perturbed genealogies, i.e. how much a single lineage choice event is affected by perturbing its downstream marker onsets (Supplementary Figure 16B,D).

Predictions are very stable for small perturbation on the order of a few hours (only 3% and 14% of predicted most likely hidden trees change in the GM and MegE datasets at $\sigma = 5$h, respectively) and the timing of the predicted lineage choice changes only little, i.e. moving the prediction from the end of a mother to the beginning of a daughter cell. This shows that exact annotation of onsets is not essential. For larger perturbation on the order of one cell cycle (12h), predictions are affected more strongly: the unperturbed hidden tree is less likely to be rediscovered and the time difference between perturbed and unperturbed prediction further increases. Note however that the predictions are still rather stable for the GM-genealogies, where large noise ($\sigma = 21h$) shifts the predicted lineage choice by less then 10h. MegE-genealogies are more affected than GM-genealogies. Here, a prediction is often based on only a few onsets and hence are more sensitive to perturbations, whereas in GM-genealogies many onsets and their intricate correlation structure inform a prediction, essentially averaging out the onset perturbations.

Supplementary Note 6: Analysis of the delay-induced correlations

The prediction of lineage choice relies on the correlated marker onsets induced by the stochastic marker delay process (Eq. 2). Naturally, the question arises: When are e.g. two marker onsets considered to be correlated due to the delay and hence downstream of the same lineage choice? To analyze the type of correlations induced by a given delay process, the following quantities are of importance (see Supplementary Figure 17A): i) The time τ from the lineage choice to the last common ancestor of the two onset cells. This is the shared history of the two onset cells. ii) The time from the last common ancestor to the respective onsets in the two cells (t_A, t_B). This is the time where the cells evolve independently of each other, starting from a perfectly correlated state at the common ancestor, to increasingly decorrelated states over time. Note that several division can happen between the common ancestor and the onsets. iii) The time difference in the two onsets $\Delta = |t_A - t_B|$.

These quantities are related via the following probability distribution:

$$P(t_A, t_B|\tau) = \sum_{x=0}^{x^*} P(x|\tau) \cdot P(t_A|x) \cdot P(t_B|x) \tag{16}$$

where x is the state of the delay process, $P(x|\tau) = P_{0\to x}(\tau)$ is the distribution of the delay process state right before the two cells split and $P(t_A|x) = \Psi_x(t_A)$ is the first passage time distribution of the delay process (similarly $P(t_B|x) = \Psi_x(t_B)$).

In the following, we will take the delay process estimated for the GM-lineage in the main paper (Fig. 3D) and analyze two aspects of Eq. 16 to gain insight about the induced correlations. For small τ, t_A and t_B are almost independent as the delay process will still be in its initial state (Supplementary Figure 17B, $\tau = 0h$). With increasing τ the delay process advances, coupling the two marker onset cells via their shared delay state $x(\tau)$ and inducing correlations in the variables t_A, t_B (Supplementary Figure 17B, $\tau = 24h$). The "pressure" for a marker onset increases (moving into the tail of the marker onset distribution) and the mean of the distribution moves towards the origin (Supplementary Figure 17B, $\tau = 36h$). Note that the process induces only weak Pearson correlations ($\max(\rho) = 0.35$ in Supplementary Figure 17B) of onset timing. Only conditioning on the last common ancestor allows a large variety of onset patterns, i.e. for $\tau = 24h$, one onset might occur two generations later ($t_A = 24h$) and its paired onset might happen only four generations later ($t_B = 48h$).

An onset shortly after cell division of the last common ancestor (i.e. small t_A or t_B) should be highly informative about the delay state in the last common ancestor, i.e. the process must be close to the state x^*. The other sister cell would inherit that state, and hence also should become marker positive soon (small Δ). Hence, we ask: What is the distribution of time difference Δ conditioned on an onset in the sister cell at t_A, i.e. the distribution $P(\Delta|t_A, \tau)$. We estimate

$P(\Delta|t_A, \tau)$ by sampling from Eq. 16. For small τ cells will be almost independent and no onsets will happen early on. However, for $\tau \geq 24h$, we observe that onsets shortly after division (small t_A) indeed produce smaller onset differences (small Δ). For example, with $\tau = 48h$, an early onset in one sister ($t_A < 10h$) will very likely produce an onset in the other sister within a 10h time window (white/yellow area in Fig. 17C, $\tau = 48h$). However, the long tails of $P(\Delta|t_A, \tau)$, as measured by the 90% quantiles (white lines in Supplementary Figure 17C) show that a large variety of onset pairs would be consistent with this delay process (every onset pair that lies below the white line). Due to these weak pair correlations one cannot simply "read off" the timepoints of lineage choice from the observed genealogies.

How can we still make reasonable predictions about the timepoint of lineage choice? Here, it is key to consider not only single pairs of onsets. Any single pair of onsets in a genealogy might be consistent with the delay process (e.g. falling into the 90% quantiles in Supplementary Figure 17B,C) and hence indicate that the two onsets derive from the same lineage choice. If we consider multiple onset pairs downstream of the same putative lineage choice and they all barely fall within the distribution, that will already indicate that the putative lineage choice is likely inconsistent with the delay process (even though each individual pair is consistent with it). Even more, one would consider not only pairs, but triplets, quadruplets, etc. to see if they are consistent with the delay process. By taking into account the entire correlation structure of onsets in a single genealogy at once, our method can reliably infer the lineage choice even in the presence of weak correlations.

Supplementary Note 7: Analysis of GMMegE genealogies

Genealogies that give rise to both GM and MegE cells are rare in the dataset of [1] (17 across three experiments) due to the tracking strategy and only scarcely tracked. Hence they were not used in estimating the model parameters from the tracked genealogies. However, they provide a test case for our prediction of lineage choice: The common ancestors of a GM-cell and a MegE cell must have been bipotent and hence lineage choice can only occur below these common ancestors. For our model, we estimated that lineage choice should predominantly happen in the first five generations (Supplementary Figure 1). The observed GMMegE genealogies support this finding (see Supplementary Figure 18 for eight exemplary GMMegE genealogies): Most GMMegE genealogies are consistent with a scenario of lineage choice in the generations 2-4, and only a single GMMegE genealogy (Supplementary Figure 18F) indicates lineage choice as late as generation 6. If instead lineage choice would predominantly occur late, one would not see the largely homogeneous (in terms of GM vs MegE) subtrees.

Supplementary References

[1] Hoppe PS et al. (2016) Early myeloid lineage choice is not initiated by random PU.1 to GATA1 protein ratios. *Nature* 535(7611):299–302.

[2] Gillespie DT (1992) A rigorous derivation of the chemical master equation. *Physica A: Statistical Mechanics and its Applications* 188(1-3):404–425.

[3] Munsky B, Khammash M (2006) The finite state projection algorithm for the solution of the chemical master equation. *The Journal of chemical physics* 124(4):044104.

[4] Van Kampen NG (1992) *Stochastic processes in physics and chemistry.* Vol. 11.

[5] Shahrezaei V, Swain PS (2008) Analytical distributions for stochastic gene expression. *Proceedings of the National Academy of Sciences* 105(45):17256–61.

[6] Aho A, Sloane N (1973) Some doubly exponential sequences. *Fibonacci Quarterly.*

[7] Bishop CM (2006) *Pattern recognition and machine learning.* (Springer-Verlag New York, Inc., New York).

[8] Huh D, Paulsson J (2011) Random partitioning of molecules at cell division. *Proceedings of the National Academy of Sciences.*

[9] Orkin SH, Zon LI (2008) Hematopoiesis: an evolving paradigm for stem cell biology. *Cell* 132(4):631–44.

[10] Huang S, Guo YP, May G, Enver T (2007) Bifurcation dynamics in lineage-commitment in bipotent progenitor cells. *Developmental biology* 305(2):695–713.

[11] Roeder I, Glauche I (2006) Towards an understanding of lineage specification in hematopoietic stem cells: a mathematical model for the interaction of transcription factors GATA-1 and PU.1. *Journal of theoretical biology* 241(4):852–65.

[12] Marr C, Strasser MK, Schwarzfischer M, Schroeder T, Theis FJ (2012) Multi-scale modeling of GMP differentiation based on single-cell genealogies. *The FEBS journal* 279(18):3488–500.

[13] Strasser MK, Theis FJ, Marr C (2012) Stability and multiattractor dynamics of a toggle switch based on a two-stage model of stochastic gene expression. *Biophysical journal* 102(1):19–29.

[14] Potoyan DA, Wolynes PG (2015) Dichotomous noise models of gene switches. *The Journal of Chemical Physics* 143(19):195101.

[15] Bialek W (2001) *Stability and noise in biochemical switches.* (The MIT Press), Vol. 13, p. 103.

[16] Gillespie DT (1976) A general method for numerically simulating the stochastic time evolution of coupled chemical reactions. *Journal of Computational Physics* 22(4):403–434.
